# “The Episodic Superhero and the Purpose of Winning”—Psychobiographer Dan McAdams on the Life and Personality of Donald Trump

**DOI:** 10.5964/ejop.4523

**Published:** 2021-08-31

**Authors:** Claude-Hélène Mayer

**Affiliations:** 1 Department of Industrial Psychology and People Management, University of Johannesburg, Johannesburg, South Africa; 2 Faculty of Cultural Studies, European University Viadrina Frankfurt (Oder), Frankfurt, Germany; University of the Free State, Bloemfontein, South Africa; Nelson Mandela University, Port Elizabeth, South Africa

**Keywords:** Donald J. Trump, book review, Dan McAdams, psychobiography, superhero, identity episodes

## Abstract

Dan P. McAdams is one of the well-known United States (US) contemporary psychobiographers and personality psychologists. Professor in psychology at Northwestern University in Evanston, Illinois and author of the recently published book, The Strange Case of Donald J. Trump: A Psychological Reckoning. McAdams has previously published psychological accounts of the lives of extraordinary political figures such as Barack Obama and George W. Bush. He specializes in life story research.

## The Chapters in the Book

This review provides an overview of the book and an insight into the life and personality of Donald J. Trump.

Initially, the title of the book reminds the reader of the famous novel of Robert Louis Stevenson, *The Strange Case of Dr Jekyll and Mr Hyde*. This 20th century novel tells the story of Dr Jekyll who suffers a personality split, induced by drinking a self-invented potion. This potion leads the doctor to split into his socially accepted public side, Dr Jekyll, and his shadow side, Mr Hyde, who becomes the protagonist.

Throughout his book, McAdams relates to Stevenson’s novel, rewriting it in the context of today’s America and the presidency of Donald Trump. The book is 305 pages long and comprises 11 chapters which follow the prologue.

In the prologue, McAdams asks himself and the reader how the story of Donald J. Trump will appear one day and how it will feel to look back in time on the divided US. He captures the current emotional and psychological split within US society about Trump and explains the origin of Trump’s primal charisma which is anchored in living and acting within the emotional moment, which is therefore experienced as authentic.

The first chapter, “Story,” begins with the statement that the “strangest thing about the story of Donald Trump is that there is no story” (McAdams, 2020, p. 8). McAdams explains that there is no coherent story about Trump, “who he really is, how he came to be, and what his life means” (McAdams, 2020, p. 8). The author puts the reader into the shoes of young Donald and selected episodes of his life and his relationship to his father, siblings and mother connecting to the construction of the myths about *life being dangerous*. Further, Donald’s experiences at a military school, his times in the 1970s and 1980s in Manhattan, his successes and growing influence and fame in society are described. McAdams explains that neither at age 38 nor age 73, does Donald J Trump have a story to tell. He has not learned to arrange a coherent pattern and sequences of his life story into a meaningful life development with overall sense-making. With insight, the author of this psychobiography explains the steps of identity development in accessible language for the reader to follow, and continuously draws conclusions about the “unpredictable presidency” (McAdams, 2020, p. 21), which leads Trump’s fans into exhilarating adventures and his critics into fear about dangerous instability.

The next chapter examines his grandfather’s history of immigration to the US in 1885, and Trump’s notion that everything in life is a “deal.” McAdams presents in a complex and well-written way the story of the male idols in the lives of Trump, his grandfather and his father, and explores the ups and downs of their business lives. He describes their glamorous deals and abilities to build wealth. The author impressively presents the five principles of deals and applies them to Trump’s family of origin, his life and his political practice in filling immediate needs, bending the rules, putting on a show, celebrating greatness and always winning. The anecdotes shared with the reader are interestingly written with detail and contextual explanations.

From an evolutionary perspective, McAdams narrates the story of rewards in world history and in Trump’s life. In so doing, he takes the reader back to an African campsite scenario 800 000 years ago, placing the reader into the shoes of *homo erectus,* facing the same challenges in the African savannah almost a million years ago as those in 21st century New York. He thereby establishes an empathy with Trump’s behaviour as “one of the most extraverted human being ever to walk the Earth” (McAdams, 2020, p. 55).

After having set the scene, McAdam’s chapter “Venom” lists a few selected victims of Donald Trump’s venomous attacks on women, “citizens of shithole countries” and selected celebrities, leading to the chapter on “Truth.” In this chapter, McAdams, as the psychobiographer, describes his attempt to put himself into the mindset of Trump and opens up the researcher–researched relationship. He deals mainly with establishing how much Trump lies, why he lies and why the supporters put up with his lies. Regarding the last question, McAdams develops five reasons which aim to explain the support Trump receives from many US citizens:

Reason #1: He doesn’t lie

Reason #2: He lies, but his emotions are true and authentic

Reason #3: He lies, but what he says “could be” true

Reason #4: He’s a con man, but he’s our con man

Reason #5: He is a different kind of being

“Love” is the focus of the next chapter in which McAdams explores the relationship between Trump and his mother, concluding that Trump’s “strange case” might not be anchored in a failed parent–son relationship. In his twenties, Trump developed a reputation as a playboy; he then married Ivana, married Marla, married Melania. The author covers the aspect of objectivation of women in Trump’s life and describes his marital relationships in-depth.

This chapter is followed by several others, such as “Me” in which McAdams describes the phenomenon of narcissism across Trump’s lifespan. This is a chapter in which the author chooses to use a more classical form of psychobiography where he explores a topic across different life sequences, from birth until the present day. Further, the “Goldwater” chapter presents Trump from a clinical perspective and explores him in the context of the narcissistic personality disorder, well aware of and connected to the Goldwater story and the Goldwater Rule (McAdams, 2020, p. 179). The Goldwater Rule keeps psychiatrists from diagnosing individuals at a distance without a personal evaluation. It was established in 1964 when nominee for president, Barry Goldwater, was publicly classified as psychologically unfit. The discourse revived in the Trump era.

The last three chapters deal with “Us” and Donald Trump’s approaches towards minorities, authorities and his aim to build a wall in the southern parts of the US bordering Mexico. The chapter “Primate” sees Trump’s ways of ruling in the context of research on “chimpanzee politics,” the story of King Kong and the influence of culture on human behaviour (McAdams, 2020, pp. 210–215). Here, McAdams presents workplace and leadership theories with regard to dominance and prestige, highlighting Trump’s approaches within the context of five points of leadership.

In the “Redemption” chapter, McAdams (2020, p. 231) concludes the story of Trump as the episodic man without “narrative beginnings and endings.” He draws conclusions about the US as a “force in the world, but *not a moral force*” (McAdams, 2020, p. 246).

## The Aim

McAdams anchors his task regarding this book in his profession as a personality psychologist:

I see *my* task as trying to understand the unique psychological makeup of the 45th president of the United States, employing the best analytic tools at my disposal and drawing upon the best research findings and evidence-based theories in psychological science. As I try to wrap my mind around the utter strangeness of Donald J. Trump my goal is to develop something sensible and psychologically illuminating to say about who he is, and how he came to be who he is. I want to understand the man. I want to explain him to you, and to myself. (McAdams, 2020, p. 184)

## McAdams’ Psychobiographical Approach

McAdams clearly fulfils his aim and the reader learns about psychological theories, different perspectives in psychology and how Trump can be understood by applying theory to his life story. Accordingly, the author involves the readership by providing various psychological perspectives, even asking readers for their opinions regarding, for example, selected items on the Right Wing Authoritarian Scale.

The book is written in an informative, catchy, well-researched and multi-perspective style. It draws on primary and secondary resources on Trump from books and articles to tweets, other social media tools, speeches, news and videos. Differing from previously scientifically written psychobiographies, this psychobiography does not present the events in the life of Donald J. Trump in chronological order, but rather builds around central themes in his family of origin, his life and his presidency. It gives insight into selected episodes of his life and his presidency and explains what contributes to Trump’s charisma and success, thereby reflecting on the common thread in his life: “without a story, but built upon episodes.” The book does not present the psychological theory in depth, but rather offers practical insights which help to explain Trump’s personality and actions.

In addition to satisfying a deep interest in understanding the 45th president of the US in more depth, the reader might also be enlightened by a practical introduction to various psychological theories to understand the personality of Trump (or others) and to experience how the author effortlessly integrates psychological and leadership theory to provide a holistic understanding of Trump as a person. However, this is not all. Dan McAdams provides the reader not only with a psychological analysis of Trump, but also with a treasure trove when it comes to understanding the presidency within the US socio-cultural context, other historical (political) figures, internal and world politics, comparisons to other political eras and the rise of authoritarian leadership and its explanation. The book provides explanations for the strong support of “Trump in contemporary US society” (McAdams, 2020, p. 191) and also examines the effects of dehumanizing language on immigrant minorities in the US.

McAdams skilfully introduces various theoretical approaches to provide the reader with tools to interpret the life and work of Donald J. Trump without clinical classifications. Through developmental and personality psychology via art and the social sciences, and relationship theories of authority, leadership, dominance and the human mind, McAdams tries to grasp the personality of Trump. He identifies parts of Trump’s life story which are built around power, success and life episodes (McAdams, 2020, p. 20) and aims to provide a non-judgmental and objective way of analysing and interpreting Trump’s life based on different perspectives. However, how can psychobiography ever provide an “objective” view of a person? McAdams uses an evidence-based approach to analyse Trump and declares in one of the later chapters by referring to Robert Louis Stevenson’s story: “I *told* you Trump was *strange.*” He then comes to the conclusion that Trump “is not Jekyll with an inner Hyde. He is, instead, all Hyde. And like Hyde, Trump projects an awesomely primal appeal. We recognize it because we are primates, too” (McAdams, 2020, p. 211).

The proposition that a person can only be Mr Hyde without also being Dr Jekyll could be counter-argued based on the positive psychology wave II (PP2.0) movement (Wong & Mayer, 2021) that a person cannot be the shadow without the light and vice versa. Therefore, McAdams’ conclusion that Trump is Mr Hyde only might be viewed as a provocative hypothesis rather than a fact-based psychological conclusion. This could be further explored by drawing on psychological theories such as PP2.0 or deeper personality investigations of “dark” personality traits (Aghababaei, Blachnio, & Lefdahl-Davis, 2021).

Throughout this psychobiography, Dan McAdams not only presents a highly intellectual and informed view on episodes in Trump’s life, but also inaugurates new ways of writing psychobiography – as an episodic art, using practically applied, multiple theoretical approaches to analyse and interpret the life of an extraordinary person. He thereby presents these theoretical chunks as easily comprehensible and valid arguments and conclusions. This makes the book easy to read and to understand since it does not draw on extensive theoretical explorations, but instead on episodes and stories to present insights into Trump’s life in the context of leadership, success, personal development and human behaviour.

Finally, this book offers a distinguishable approach to psychobiography and expands previous new approaches (Mayer & Köváry, 2019). It will be of great interest to anyone who wishes to decipher the complex personality of Donald J Trump in new ways.
